# Machine Learning‐Based Prediction of Mortality in Patients With Mitral Regurgitation: Results From Two Nationwide, Prospective Studies

**DOI:** 10.1002/mco2.71005

**Published:** 2026-10-09

**Authors:** Yuzhe Yin, Junxing Lv, Qinghao Zhao, Yunqing Ye, Weiwei Wang, Haitong Zhang, Zhenyan Zhao, Qingrong Liu, Zikai Yu, Bin Zhang, Shuai Guo, Zhenya Duan, Bincheng Wang, Yanyan Zhao, Runlin Gao, Zhe Li, Haiyan Xu, Yongjian Wu, Junbo Ge, for the CHINA‐VHD collaborators

**Affiliations:** ^1^ Department of Cardiology Fuwai Hospital National Center for Cardiovascular Diseases Chinese Academy of Medical Sciences and Peking Union Medical College Beijing China; ^2^ Department of Cardiology Peking University People's Hospital Beijing China; ^3^ Medical Research & Biometrics Center Fuwai Hospital National Center for Cardiovascular Diseases Chinese Academy of Medical Sciences and Peking Union Medical College Beijing China; ^4^ Department of Cardiology Zhongshan Hospital Shanghai Institute of Cardiovascular Diseases Fudan University Shanghai China

**Keywords:** CatBoost, external validation, machine learning, mitral regurgitation, mortality prediction, SHAP analysis

## Abstract

Mitral regurgitation (MR) is among the most prevalent valvular heart diseases. We developed and externally validated a machine learning (ML)‐based mortality prediction model for patients with significant MR. From the ChinaValvular Heart Disease (China‐VHD) registry, 6323 patients with moderate or greater MR were randomly split 7:3 into training and internal validation sets. Variables selected concordantly by Cox stepwise regression, Least Absolute Shrinkage and Selection Operator (LASSO)‐penalized Cox regression, and Boruta were retained. Seventeen ML and traditional models were compared. The final model was tested in the internal set and an external cohort of 4658 patients from the China Elderly Valve Disease (China‐DVD) study. SHapley Additive exPlanations (SHAP) analysis assessed interpretability. Fifteen predictors were identified. CatBoost performed best, with AUCs of 0.783 (95% confidence interval [CI] 0.751–0.815) internally and 0.703 (0.678–0.728) externally, outperforming Cox (0.699), MitraScore (0.620), and EuroSCORE II (0.679). Age, NYHA class, and left ventricular ejection fraction were the top predictors. The model effectively distinguished high‐risk from low‐risk patients internally (adjusted hazard ratio [HR] 5.49, *p* < 0.001) and externally (adjusted HR 3.05, *p* < 0.001). In conclusion, we developed and externally validated an ML‐based mortality prediction model for MR irrespective of etiology or treatment strategy, with potential to improve risk stratification and clinical decision‐making.

## Graphical abstract

Using two nationwide prospective Chinese cohorts totaling nearly 11,000 patients with significant mitral regurgitation, we developed and externally validated a CatBoost‐based machine learning model for all‐cause mortality prediction. The model achieved AUCs of 0.783 internally and 0.703 externally, outperforming the Cox model, MitraScore, and EuroSCORE II. It enables effective risk stratification and is freely accessible via the MitraBoost web calculator.

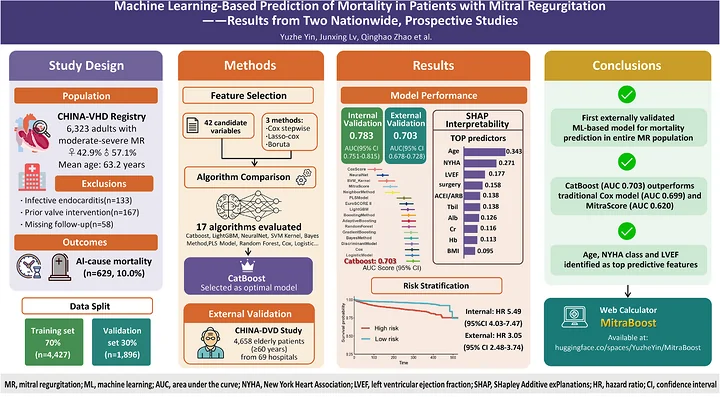

## 1 Introduction

Mitral regurgitation (MR) is one of the most prevalent valvular heart diseases (VHD) globally [1, 2]. For patients with significant MR, accurate prognostic assessment is paramount for guiding critical therapeutic decisions, such as the optimal timing for surgical or transcatheter intervention. In compliance with the guidelines of the management of VHD, contemporary clinical practice predominantly relies on the severity of heart failure symptoms and a set of echocardiographic parameters. These indices, while valuable, possess inherent limitations in capturing the complex, nonlinear interactions between comorbidities, cardiac remodeling, and functional status that collectively determine the outcome of MR patients.

Furthermore, the landscape of MR management is evolving, underscored by divergent results from major randomized controlled trials investigating the efficacy of mitral valve transcatheter edge‐to‐edge repair (M‐TEER) [3, 4, 5]. These landmark findings highlight the urgent clinical need for more effective tools to identify patients at different levels of mortality risk when minimally invasive procedures for MR are considered. An accurate, individualized risk prediction model could help address this uncertainty, potentially informing personalized therapeutic pathways.

The advent of machine learning (ML) provides a transformative paradigm for prognostic modeling in cardiovascular medicine [6, 7, 8, 9]. Unlike traditional statistical approaches that often rely on pre‐specified linear assumptions, ML algorithms can automatically learn complex patterns from high‐dimensional, real‐world clinical data. Among various ML techniques, ensemble methods like gradient boosting machines have demonstrated particular prowess in predictive accuracy [7, 8, 9]. However, the application of ML specifically for the prediction of mortality in patients with MR remains nascent. Two existing models only focus on patients treated by M‐TEER, lack large‐scale external validation, and have not undergone comprehensive benchmarking against a wide array of alternative ML algorithms [8, 9]. The absence of a robust, generalizable model significantly hinders the clinical translation of ML‐based prognostication to patients with significant MR.

Leveraging comprehensive data from two nationwide prospective cohorts, the present study aimed to identify the most performant machine‐learning algorithm for the prediction of mortality in MR. On the basis of the selected optimal model, we sought to establish and externally validate a pragmatic prognostic instrument for patients with MR, augmenting it with interpretative analysis and an online calculator to bridge the gap between prediction and clinical application.

## 2 Results

### 2.1 Overall Study Cohort

A total of 6323 patients with moderate or greater MR from the China Valvular Heart Disease (China‐VHD) registry were randomly divided into a training set (70%, *n* = 4427) and an internal validation cohort (30%, *n* = 1896). Patients (*n* = 4658) from the China Elderly Valve Disease (China‐DVD) registry served as the external validation cohort (Figure 1).

**FIGURE 1:**
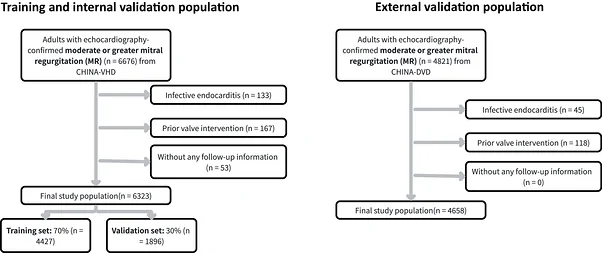
Flowchart of cohort selection.

Baseline characteristics were summarized in Table 1. Patients in the China‐VHD cohort had a mean age of 63.2 ± 12.8 years with 57.1% male patients, whereas those from the China‐DVD cohort were older (70.7 ± 7.4 years), and 2435 (52.3%) were male. The median follow‐up durations were 733 and 365 days for the China‐VHD and China‐DVD cohorts, respectively. During follow‐up, 629 deaths (10.0%) occurred in China‐VHD and 460 deaths (9.9%) in China‐DVD. The 2‐year cumulative survival rates were 0.89 (95% confidence interval [CI], 0.88–0.90) for the training set and 0.89 (95% CI, 0.87–0.91) for the internal validation set, whereas the 1‐year survival rate in the external validation cohort was 0.89 (95% CI, 0.88–0.90), with no significant difference among cohorts (*p* = 0.991).

**TABLE 1:** Baseline characteristics of the study population.

Variable	China‐VHD cohort				China‐DVD cohort	*p* value^a^
	Overall (*n* = 6323)	Training set (*n* = 4427)	Internal validation set (*n* = 1896)	*p* value^b^	External validation set (*n* = 4658)	
Demographics						
Age, mean (SD), years	63.18 (12.78)	63.38 (12.77)	62.71 (12.81)	0.058	70.68 (7.41)	<0.001
Sex, male (%)	3611 (57.1)	2549 (57.6)	1062 (56.0)	0.261	2435 (52.3)	<0.001
Smoking history (%)	4146 (65.6)	2892 (65.3)	1254 (66.1)	0.530	3277 (70.4)	<0.001
Physical examination						
BMI, mean (SD), kg/m^2^	23.63 (3.65)	23.67 (3.71)	23.53 (3.50)	0.173	23.36 (3.58)	<0.001
SBP, mean (SD), mmHg	124.50 (20.42)	124.44 (20.64)	124.62 (19.91)	0.758	129.08 (20.94)	<0.001
DBP, mean (SD), mmHg	74.78 (13.17)	74.88 (13.34)	74.54 (12.77)	0.338	75.38 (12.97)	0.037
Heart rate, mean (SD), bpm	79.56 (18.00)	79.50 (18.02)	79.70 (17.97)	0.683	80.21 (18.44)	0.172
Laboratory tests						
Hemoglobin, mean (SD), g/L	131.37 (20.17)	131.14 (20.27)	131.90 (20.01)	0.166	124.67 (25.35)	<0.001
Serum creatinine, mean (SD), µmol/L	93.92 (63.61)	95.44 (67.71)	90.37 (52.64)	0.004	97.79 (67.11)	<0.001
eGFR, mean (SD), mL/min/1.73m^2^	79.62 (28.26)	79.00 (28.48)	81.08 (27.68)	0.007	83.48 (221.86)	<0.001
Albumin, mean (SD), g/L	39.06 (5.04)	39.09 (5.10)	39.01 (4.91)	0.576	38.62 (7.52)	0.001
Total bilirubin, mean (SD), µmol/L	18.68 (14.58)	18.67 (14.19)	18.68 (14.74)	0.979	19.18 (15.86)	0.291
Echocardiography						
LA, mean (SD), mm	48.27 (9.61)	48.26 (9.43)	48.29 (9.92)	0.916	47.22 (12.19)	<0.001
LVEDD, mean (SD), mm	57.46 (10.47)	57.58 (10.49)	57.19 (10.43)	0.175	56.86 (17.29)	0.046
LVEF, mean (SD), %	49.81 (14.55)	49.64 (14.56)	50.21 (14.51)	0.152	50.23 (14.25)	0.111
Clinical classification						
NYHA I (%)	75 (1.6)	53 (1.6)	22 (1.6)	0.182	376 (9.2)	<0.001
NYHA II (%)	1652 (35.4)	1143 (35.2)	509 (37.5)		1095 (26.8)	
NYHA III (%)	2021 (43.3)	1423 (43.8)	598 (44.0)		1782 (43.7)	
NYHA IV (%)	861 (18.4)	632 (19.4)	229 (16.9)		828 (20.3)	
Comorbidities						
Hypertension (%)	2944 (46.6)	2076 (46.9)	868 (45.8)	0.432	2504 (53.8)	<0.001
Hyperlipidemia (%)	848 (13.4)	613 (13.8)	235 (12.4)	0.130	313 (6.7)	<0.001
Diabetes mellitus (%)	1099 (17.4)	784 (17.7)	315 (16.6)	0.309	1043 (22.4)	<0.001
Coronary artery disease (%)	2373 (37.5)	1690 (38.2)	683 (36.0)	0.112	1737 (37.3)	0.261
Myocardial infarction (%)	831 (13.1)	602 (13.6)	229 (12.1)	0.110	621 (13.3)	0.252
Cardiomyopathy (%)	785 (12.4)	549 (12.4)	236 (12.4)	0.993	505 (10.8)	0.041
Atrial fibrillation (%)	1982 (31.3)	1404 (31.7)	578 (30.5)	0.349	1678 (36.0)	<0.001
Pacemaker implantation (%)	485 (7.7)	340 (7.7)	145 (7.6)	1.000	305 (6.5)	0.079
Peripheral artery disease (%)	162 (2.6)	109 (2.5)	53 (2.8)	0.496	205 (4.4)	<0.001
Stroke (%)	584 (9.2)	425 (9.6)	159 (8.4)	0.139	524 (11.2)	0.001
COPD (%)	381 (6.0)	264 (6.0)	117 (6.2)	0.795	235 (5.0)	0.083
Chronic kidney disease (%)	447 (7.1)	341 (7.7)	106 (5.6)	0.003	378 (8.1)	0.002
Cancer (%)	100 (1.6)	75 (1.7)	25 (1.3)	0.324	107 (2.3)	0.015
Valvular diseases						
MR				0.357		<0.001
Moderate (%)	3971 (62.8)	2797 (63.2)	1174 (61.9)		3188 (68.4)	
Severe (%)	2352 (37.2)	1630 (36.8)	722 (38.1)		1470 (31.6)	
Significant AR (%)	1053 (16.7)	731 (16.5)	322 (17.0)	0.672	813 (17.5)	0.476
Significant AS (%)	267 (4.2)	185 (4.2)	82 (4.3)	0.844	240 (5.2)	0.071
Significant MS (%)	528 (8.4)	346 (7.8)	182 (9.6)	0.021	319 (6.8)	0.001
Significant TR (%)	2489 (39.4)	1763 (39.8)	726 (38.3)	0.265	1835 (39.5)	0.515
Pulmonary hypertension (%)	2896 (45.8)	2037 (46.0)	859 (45.3)	0.624	2178 (46.8)	0.534
Etiology						
Primary MR (%)	3070 (48.6)	2166 (48.9)	904 (47.7)	0.398	1925 (41.8)	<0.001
Secondary MR (%)	3253 (51.4)	2261 (51.1)	992 (52.3)		2675 (58.2)	
Procedures						
PCI (%)	1018 (16.1)	731 (16.5)	287 (15.1)	0.185	628 (13.5)	<0.001
CABG (%)	358 (5.7)	234 (5.3)	124 (6.5)	0.055	312 (6.7)	0.013
Surgical valve intervention (%)	1172 (18.5)	790 (17.8)	382 (20.1)	0.034	861 (18.5)	0.097
Transcatheter procedure (%)	63 (1.0)	37 (0.8)	26 (1.4)	0.068	125 (2.7)	<0.001
Medications						
Beta‐blockers (%)	3952 (62.5)	2776 (62.7)	1176 (62.0)	0.628	2905 (62.4)	0.868
Diuretics (%)	4899 (77.5)	3443 (77.8)	1456 (76.8)	0.411	3566 (76.6)	0.365
ACEI/ARB (%)	3245 (51.3)	2261 (51.1)	984 (51.9)	0.566	2353 (50.5)	0.589
Outcome						
Mortality (%)	629 (10.0)	441 (10.0)	188 (9.9)	0.955	460 (9.9)	0.991
Survival time, mean (SD), days	553.20 (272.26)	535.43 (271.26)	600.06 (273.94)	<0.001	295.79 (128.79)	<0.001

Abbreviations: ACEI/ARB, angiotensin‐converting enzyme inhibitor/angiotensin receptor blocker; AF, atrial fibrillation; Alb, albumin; AR, aortic regurgitation; AS, aortic stenosis; BMI, body mass index; CABG, coronary artery bypass grafting; CAD, coronary artery disease; China‐DVD, China elderly valve disease; China‐VHD, China valvular heart disease; CKD, chronic kidney disease; CM, cardiomyopathy; COPD, chronic obstructive pulmonary disease; Cr, creatinine; DBP, diastolic blood pressure; eGFR, estimated glomerular filtration rate; Hb, hemoglobin; HR, heart rate; LA, left atrium diameter; LVEDD, left ventricular end‐diastolic diameter; LVEF, left ventricular ejection fraction; MI, myocardial infarction; MR, mitral regurgitation; MS, mitral stenosis; NYHA, New York Heart Association functional class; PAD, peripheral arterial disease; PCI, percutaneous coronary intervention; PM, pacemaker; SBP, systolic blood pressure; SD, standard deviation; Tbil, total bilirubin; TR, tricuspid regurgitation.

^a^ *p* value for comparison among the training, internal validation, and external validation sets.

^b^ *p* value for comparison between training and internal validation sets within China‐VHD cohort.

### 2.2 ML Models for Mortality Prediction

CatBoost showed the best discrimination in both internal and external validation datasets (Figures 2A,B and 3A–C). A threshold of 0.103 derived in the development cohort based on the maximum Youden index (sensitivity 0.794 and specificity 0.753) was subsequently applied to both the internal and external validation datasets. In the internal validation set, CatBoost achieved an AUC of 0.783 (95% CI, 0.751–0.815), a sensitivity of 0.676, and a specificity of 0.756. In the external validation set, the AUC of CatBoost was 0.703 (95% CI, 0.678–0.728), with a sensitivity of 0.698 and a specificity of 0.588. Its performance exceeded that of the traditional Cox model (AUC 0.699) and markedly outperformed MitraScore (AUC 0.620), CoxScore (AUC 0.564) and EuroSCORE II (AUC 0.679). The Brier Scores of CatBoost were 0.0814, 0.0685, and 0.0875 in the training, internal validation, and external validation sets, respectively, comparable to other top‐performing models and substantially lower than EuroSCORE II and traditional scoring systems (Table S6). BIC and DeLong test comparisons between CatBoost and the Cox model are provided in Table S7. CatBoost was therefore selected as the final model for subsequent analyses.

**FIGURE 2:**
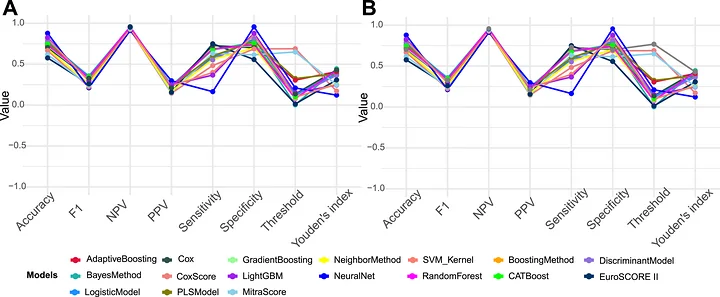
Comparative performance metrics across predictive models in the internal and external validation sets. (A) Internal validation set performance metrics and (B) external validation set performance metrics. NPV, negative predictive value; PPV, positive predictive value.

**FIGURE 3:**
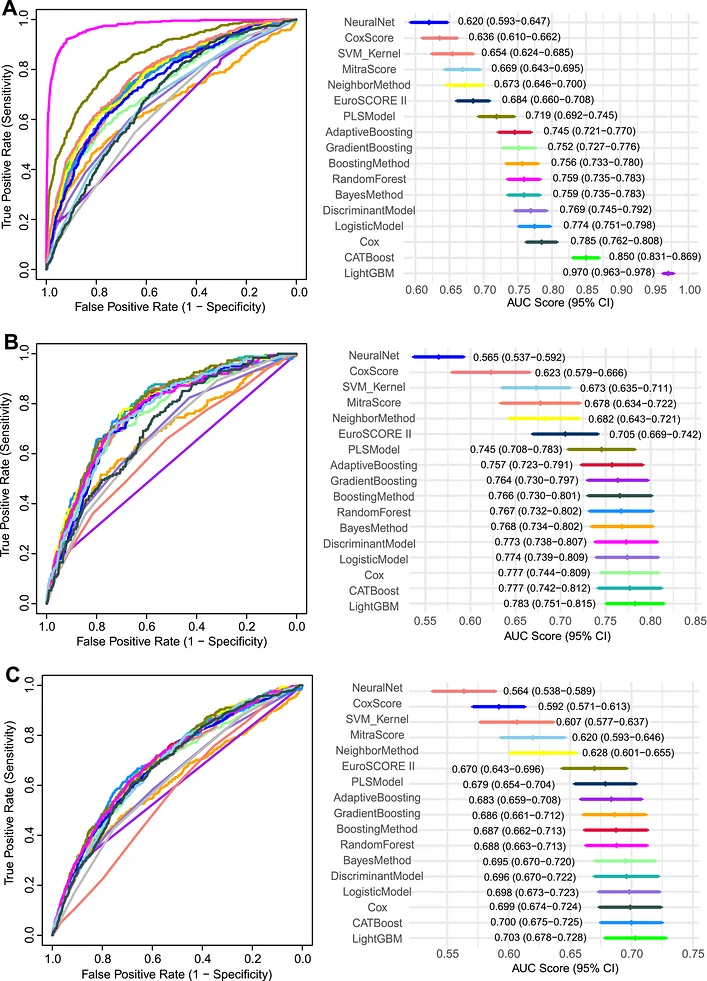
Comparison of different ML methods and currently available risk scores. (A) Training set ROC curves and forest plot of each model AUC score; (B) internal validation set ROC curves and forest plot of each model AUC score; and (C) external validation ROC curves and forest plot of each model AUC score. AUC, area under the curve; ROC, receiver operating characteristic.

### 2.3 Risk Stratification and Clinical Utility

Patients were stratified into high‐ and low‐risk groups on the basis of the CatBoost‐predicted probabilities. Kaplan–Meier analysis revealed a significant divergence in survival between risk groups in both the internal and external validation cohorts (Figure 4A,B). In the internal validation set, high‐risk patients exhibited a markedly elevated risk of mortality (adjusted hazard ratio [HR] = 5.49, 95% CI 4.03–7.47; *p* < 0.001; log‐rank *p* < 0.0001). This finding was corroborated in the external validation set, where the high‐risk group maintained a significantly higher hazard compared with those in the low‐risk group (adjusted HR = 3.05, 95% CI 2.48–3.74; *p* < 0.001; log‐rank *p* < 0.0001).

**FIGURE 4:**
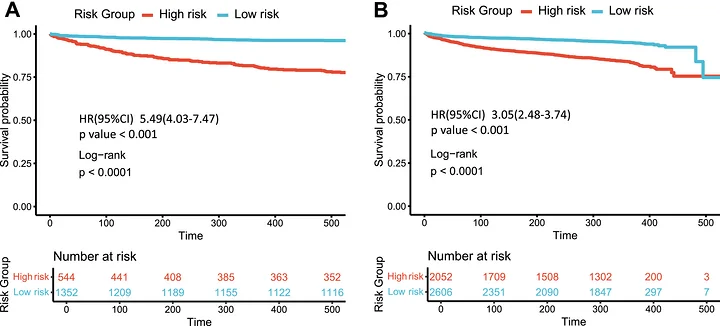
Kaplan–Meier survival curves stratified by predicted risk group. (A) Internal validation population and (B) external validation population. CI, confidence interval; HR, hazard ratio.

Clinical impact curves were plotted to assess the clinical applicability of the CatBoost model (Figure 5A,B). At lower thresholds, the number of patients classified as high risk substantially exceeded the number of observed deaths; as the threshold increased, the two curves converged and became nearly superimposed above approximately 0.5, indicating a progressively decreasing false‐positive burden. Calibration curves demonstrated good agreement between predicted and observed mortality across all three datasets (Figure 5C–E). Additional performance metrics, including confusion matrices and residual distributions, were provided in Figures S1 and S2A–C.

**FIGURE 5:**
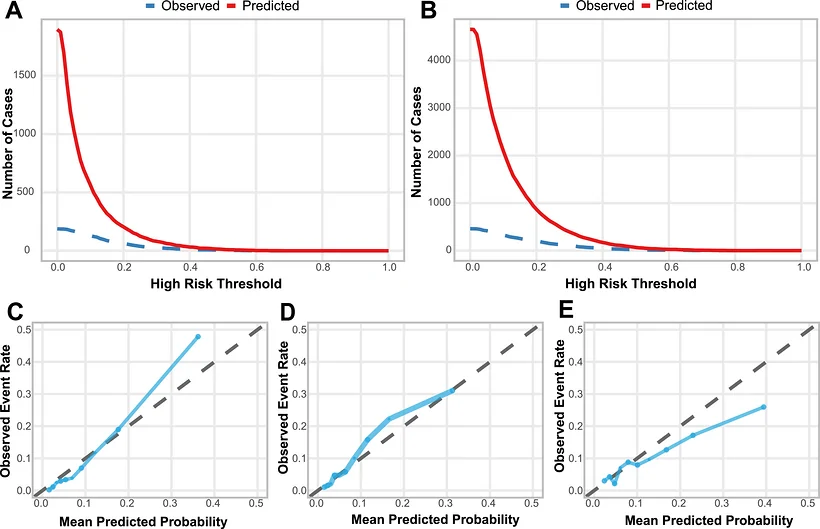
Clinical utility and calibration of the CatBoost model in the internal and external validation datasets. Clinical impact curves in the (A) internal and (B) external validation sets; calibration curves in the (C) training, (D) internal validation, and (E) external validation sets.

To enhance research reproducibility and model utility, the trained CatBoost model has been deployed on the Hugging Face Spaces platform (https://huggingface.co/spaces/YuzheYin/MitraBoost). The application is publicly accessible without the need for additional software installation, providing a convenient validation tool for subsequent research and clinical applications. Representative screenshots of the user interface, including example inputs and the corresponding predicted risk outputs for two illustrative cases, are provided in Figure S10A,B.

### 2.4 Model Interpretability Using SHapley Additive exPlanations (SHAP)

The contribution of individual features to the CatBoost model's predictions was interpreted using the SHAP analysis (Figure 6A). As assessed by the mean absolute SHAP value, age was identified as the most influential predictor (mean |SHAP| = 0.343), followed by NYHA class (0.271), left ventricular ejection fraction (LVEF) (0.177), valve surgery (0.158), the use of ACEI/ARB (0.138), and total bilirubin (0.138).

**FIGURE 6:**
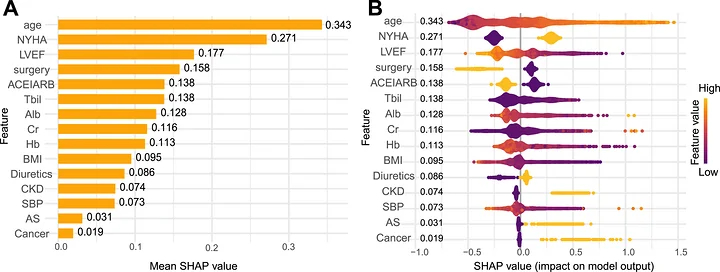
SHAP analysis for the CatBoost model. (A) Feature importance ranked by mean absolute SHAP value. (B) SHAP summary plot illustrating the relationship between feature values and their impact on mortality prediction. Red indicates high feature values; blue indicates low feature values. ACEI/ARB, angiotensin‐converting enzyme inhibitor/angiotensin receptor blocker; AS, significant aortic stenosis; BMI, body mass index; CKD, chronic kidney disease; SHAP, SHapley Additive exPlanations; LVEF, left ventricular ejection fraction; NYHA, New York Heart Association functional class (III/IV vs. I/II); SBP, systolic blood pressure; Tbil, total bilirubin.

The SHAP summary plot (Figure 6B) demonstrated that higher predicted mortality risk was associated with older age, higher NYHA class, reduced LVEF, the absence of valve surgery, lack of ACEI/ARB therapy, and higher levels of bilirubin and creatinine, as well as lower albumin, lower hemoglobin, and the presence of CKD. Conversely, valve surgery and the use of ACEI/ARB were associated with lower predicted mortality risk. Feature dependence plots and individual case explanations were shown in Figures S3 and S4.

### 2.5 Subgroup and Sensitivity Analyses

Subgroup analyses confirmed consistent model performance across clinically relevant subsets, including patients with primary or secondary MR, NYHA class II–IV, LVEF ≤ 60%, and those receiving conservative treatment. ROC curves for each subgroup are presented in Figure S5A–E, and a forest plot summarizing AUCs with 95% CIs across all subgroups is provided in Figure S6.

A full‐variable CatBoost model using all 42 candidate variables achieved AUCs of 0.859 (training), 0.780 (internal validation), and 0.702 (external validation), showing no meaningful improvement over the 15‐variable model (Figure S7). The SHAP analysis of the full model yielded a similar variable ranking, with age and NYHA class remaining the most contributive predictors (Figure S8).

Time‐dependent ROC analysis demonstrated stable discrimination of CatBoost across follow‐up landmarks, with AUCs of 0.859 and 0.861 at 6 and 12 months in the training set, 0.777 and 0.777 in the internal validation set, and 0.711 and 0.704 in the external validation set, respectively (Figure S9).

## 3 Discussion

Leveraging data from two large, nationally representative cohorts of Chinese patients with moderate or greater MR, we developed and externally validated a comprehensive ML‐basedmodel for predicting long‐term mortality. After careful evaluation of 17 ML and traditional models, CatBoost emerged as the optimal model, demonstrating superior discrimination in both internal (AUC 0.783) and external validation (AUC 0.703) cohorts. The SHAP‐based interpretability analysis revealed age, NYHA functional class, and LVEF as the most influential mortality predictors. This study provides a nationwide, externally validated machine‐learning model for mortality prediction in patients with significant MR, irrespective of etiology or treatment strategy, offering a practical framework for precision risk stratification and individualized therapeutic decision‐making in routine clinical practice.

Traditional risk prediction models for the prognostication of MR have predominantly relied on the Cox or logistic regression, with a series of intractable deficiencies. For instance, conventional surgical risk scores (EuroSCORE II and STS score) demonstrated limited discriminatory capacity in MR populations, and MR‐specific models, like MIDA‐Q, remained etiology‐restricted to mitral valve prolapse [10]. The MitraScore [11] employed Cox regression to predict 1‐year mortality risk after transcatheter edge‐to‐edge repair (TEER), achieving *c*‐statistics of only 0.70 and 0.66 in derivation and validation cohorts. Indeed, the traditional statistical approaches fundamentally assume linear relationships between predictors and outcomes, inadequately capturing the complex, nonlinear pathophysiological dynamics and multifactorial interactions—that determine the progression of MR as well as long‐term prognosis.

Recent ML applications in MR have shown continuous progress but are largely confined to specific interventional contexts. The EuroSMR Risk Score [8] advanced the field by applying XGBoost with 18 parameters to 4600 secondary MR patients undergoing TEER, achieving superior discrimination (validation AUC 0.789) and successfully identifying extreme‐risk patients with >70% predicted 1‐year mortality. Similarly, the MITRALITY score [9] utilized XGBoost with six metabolic and hemodynamic parameters (urea, hemoglobin, NT‐proBNP, creatinine, BMI, and mean arterial pressure) in 1009 TMVR patients, demonstrating excellent performance (validation AUC 0.783) and outperforming GWTG (AUC 0.721), GRASP (AUC 0.657), and EuroSCORE (AUC 0.616). Although these ML‐based scores represent important methodological advances in applying sophisticated algorithms—including recursive feature elimination, SHAP‐based interpretability, and Platt calibration, they exclusively focused on highly selected procedural populations, specifically, patients who were already considered suitable for TEER or TMVR. The inherent complexity of MR, characterized by diverse pathophysiological mechanisms and varying clinical presentations, necessitates a more comprehensive approach for risk stratification that extends beyond interventional suitability [12]. To address these gaps, we developed and externally validated a comprehensive ML‐based model using two large‐scale, nationally representative cohorts encompassing a broad, nonselected population of significant MR, irrespective of etiology or treatment strategy. Through systematic comparisons of 12 machine learning algorithms which included XGBoost, the CatBoost model demonstrated optimal performance with robust discrimination across clinically relevant subgroups. Importantly, the predictive value of the CatBoost model was maintained in an independent validation cohort with heterogeneous patient characteristics, and its interpretability was unraveled by the SHAP analysis. As a representative of traditional risk scoring system, the MitraScore achieved an AUC of only 0.620 in the present MR population, substantially below the currently developed model (internal validation AUC 0.783; external validation AUC 0.703). Notably, the added value of the ML approach resides not in the identification of novel predictors per se, but in the capacity to model complex nonlinear relationships and high‐order interactions that traditional linear models cannot capture. The SHAP dependence plots revealed threshold‐dependent and nonlinear effects of several key variables, and the framework further enabled individualized risk profiling at the patient level, representing functionalities that are not readily achievable with conventional Cox regression.

In the present study, the SHAP analysis provided clinically relevant insights into the individual contributions of predictive features. Age emerged as the most influential predictor, reflecting its fundamental impact in chronic MR. Aging independently affects left ventricular remodeling patterns, with older patients exhibiting distinct structural and functional adaptations compared to younger individuals, even in the absence of other significant comorbidities [13]. NYHA class demonstrated substantial predictive value as a comprehensive measure of symptom burden and functional limitation. Analysis from the COAPT trial showed that rates of death or heart failure hospitalization increased progressively with higher NYHA class, with the classification providing robust prognostic utility across the spectrum of secondary MR severity [14]. Left ventricular dysfunction, as mirrored by LVEF, portends worse outcomes regardless of intervention strategy, with the critical threshold of ≤60% serving as a Class I indication for surgical intervention in asymptomatic primary MR according to 2021 European Society of Cardiology (ESC)/European Association for Cardio‐Thoracic Surgery (EACTS) and 2020 American College of Cardiology (ACC)/American Heart Association (AHA) guidelines [15, 16]. Our analysis also identified lower BMI as an independent predictor of mortality, consistent with previous observations in MR populations [17], reflecting the close relationship between frailty, malnutrition, and adverse outcomes in cardiovascular populations [18]. The strong predictive impact of serum creatinine and chronic kidney disease supported the prognostic role of renal impairment in MR [19] implying the impact of cardio–renal interaction in MR pathophysiology. Indeed, the recent EFFORT trial demonstrated that ertugliflozin, the agent with cardio–renal protective effects, significantly could reduce functional MR in patients with heart failure and mildly to moderately reduced ejection fraction, accompanied by improvements in left ventricular global longitudinal strain and left atrial remodeling [20]. Total bilirubin and albumin levels also contributed independently to outcome prediction, reflecting the significance of hepatic congestion and systemic inflammation as documented in large‐scale heart failure studies [21, 22]. These findings highlight the hepato–cardiac axis in advanced MR pathophysiology [6]. Concomitant aortic stenosis was identified as an adverse prognostic factor, consistent with registry data demonstrating that combined valvular lesions nearly double mortality risk compared with isolated MR [23]. As for medications, the use of diuretics was a significant marker of advanced heart failure [24], as well as VHD with poor prognosis [25]. Conversely, ACEI/ARB use was associated with lower predicted mortality, consistent with evidence from the PRIME trial suggesting improvement of secondary MR through renin–angiotensin system inhibition [26]. However, this association likely reflects both pharmacological benefit and treatment selection, as patients with better functional status and fewer comorbidities are more likely to receive these agents. Surgical valve intervention, the hemoglobin level, and systolic blood pressure also contributed to model performance, capturing additional dimensions of clinical complexity. Altogether, the present multidimensional system integrates both guideline‐endorsed variables and nontraditional biomarkers, which allows our model to capture the intricate interplay of factors influencing mortality in MR patients and provides a more nuanced risk assessment that reflects the systemic nature of advanced valvular disease and offers potential insights for therapeutic target identification.

Our findings carried several crucial implications for clinical risk stratification and therapeutic decision‐making of MR. First, previous randomized controlled trials evaluating TEER for secondary MR yielded divergent results [3, 4, 5]. Studies based on real‐world data also showed that although mitral valve intervention substantially improved survival, mortality risk remained considerable, and a substantial proportion of patients might not derive benefit from the procedure [27]. These observations underscore the critical challenge of prospectively identifying which patients will derive a sustained survival benefit from intervention, as opposed to those for whom medical therapy remains preferable or for whom intervention may be too late. Our prognostic model addresses this unmet need by providing an individualized risk estimate to inform patient‐specific therapeutic pathways. Second, moving beyond prediction, our model feature exploration identified several prognostic factors that might represent potential therapeutic targets. The prognostic importance of renal function and hepatic congestion markers suggests that medical optimization of these extra‐organ systems can be a vital adjunct to valve‐directed procedures. Third, from an implementation standpoint, our model's reliance on routinely available clinical parameters ensures its broad applicability without imposing additional cost or testing burden. The development of the current online calculator, and the potential for the integration of the model into electronic health systems, paves the way for automated, efficient, and precision‐guided care in MR management. From a practical standpoint, the model outputs an individualized mortality probability that can guide clinical management at multiple levels. Patients stratified as high‐risk may benefit from intensified surveillance of cardiac, renal, and hepatic function, earlier consideration of surgical or transcatheter intervention, and proactive optimization of modifiable risk factors identified by SHAP analysis—including anemia, renal impairment, and neurohormonal blockade. The publicly available online calculator further enables seamless integration of the model into routine clinical workflows.

### 3.1 Limitations

Several limitations should be acknowledged. First, the model was developed and validated exclusively in Chinese populations, and generalizability to other ethnic groups requires further investigation. Second, predictive performance was constrained by the variables captured in the source databases; NT‐proBNP and detailed valvular anatomy were not uniformly available, precluding direct comparison with models that incorporate these indices [8, 9]. Incorporating emerging disease‐specific biomarkers and advanced imaging parameters may further enhance predictive accuracy in subsequent iterations. Third, the external validation AUC of 0.703 indicates modest discrimination, likely reflecting substantial heterogeneity between the development and validation cohorts in age, comorbidity burden, and follow‐up duration. This performance is nonetheless comparable to or better than existing externally validated models in valvular heart disease, such as MitraScore (*c*‐statistics of 0.66 and 0.614 in independent cohorts) [11, 28]; available benchmark comparators also have intrinsic constraints, as MitraScore was originally developed for M‐TEER recipients rather than the general MR population [11], and EuroSCORE II targets perioperative rather than long‐term risk [29]. External validation in cohorts with follow‐up beyond 12 months will further clarify long‐term prognostic utility. Fourth, the study period predated both the widespread adoption of transcatheter mitral interventions and the routine use of SGLT2 inhibitors, the latter of which have been shown to reduce functional MR [20]; future work should reassess model performance in contemporary cohorts reflecting these therapeutic shifts. Fifth, because the “poor mobility” item was unavailable in our databases, EuroSCORE II was calculated without it, which may have modestly underestimated its performance and should be considered when interpreting the model comparison.

## 4 Conclusions

We developed and externally validated a robust ML model based on the CatBoost algorithm that accurately predicted mortality risk in patients with significant MR. The model outperformed a wide array of alternative algorithms and demonstrated generalizability in an independent validation cohort. By leveraging routinely collected clinical data, it provided a powerful, automated approach for prospective risk stratification. The implementation of this instrument holds promise for enhancing prognostic evaluation and personalizing management strategies for patients with MR, ultimately aiming to improve clinical outcomes.

## 5 Materials and Methods

### 5.1 Study Population

The patient population for model development and internal validation was from the China‐VHD registry, a prospective, multicenter, nationwide, observational cohort study of adults (≥18 years) with echocardiographically confirmed significant (moderate or greater) VHD. From April to June 2018, 13,917 patients were consecutively enrolled from 46 medical centers in China. The design and quality control measures of the China‐VHD study were published previously [6, 30]. The study was performed in accordance with the ethical principles of the Declaration of Helsinki and was granted approval by the institutional review board of the participating institution. All participants provided written informed consent. After excluding individuals with infective endocarditis, prior valvular intervention, or without any follow‐up information, 6323 patients with significant MR constituted the model development and internal validation sets (Figure 1).

For the external validation of the model, we utilized data from the China‐DVD study. From September to December 2016, a total of 8929 elderly inpatients (≥60 years) with significant VHD were recruited from 69 large academic hospitals [31]. The study was conducted in accordance with the Declaration of Helsinki, with approval from all participating centers’ institutional review boards or ethics committees and written informed consent from all participants. Following the application of the same exclusion criteria with the development cohort, 4658 patients constituted the external validation population. Study size was determined by the number of eligible participants in the two existing registries; all eligible participants with available follow‐up were included.

### 5.2 Echocardiography

Transthoracic echocardiography with two‐dimensional and Doppler imaging was performed for all participants using standard ultrasound systems in both China‐VHD and China‐DVD studies. Cardiac chamber dimensions were assessed in line with the recommendations from the American Society of Echocardiography and the European Association of Cardiovascular Imaging [32]. LVEF, derived via the biplane modified Simpson's method, served as the measure of left ventricular systolic function. The echocardiographic definitions for significant MR and the measures implemented for the quality control of echocardiography are provided in the Supporting Information section and have been described in our previous publications [25, 30, 31].

### 5.3 Follow‐Up and Endpoint

In the China‐VHD study, patients were followed up at 6, 12, 18, and 24 months via clinical visits, telephone interviews, or medical record review. The present analysis focused on all‐cause mortality as the prediction outcome. Regarding the China‐DVD study, its follow‐up procedures and data collection have been previously reported, as detailed in prior publications [31, 33].

### 5.4 Feature Selection

A total of 42 candidate variables were initially considered for model construction, encompassing demographic characteristics, clinical features, laboratory parameters, echocardiographic measurements, comorbidities, and treatment modalities. Cox stepwise regression, Least Absolute Shrinkage and Selection Operator (LASSO)‐penalized Cox model, and the Boruta algorithm were used to identify the most predictive variable subset and minimize overfitting while enhancing clinical applicability. Only features consistently selected as prognostically relevant by all three methods were retained. This rigorous selection process yielded 15 predictive variables for subsequent development of the ML model, with univariate and multivariable Cox regression analyses summarized in Table 2. Variable screening results by the three methods were shown in Table S1. Variance inflation factor (VIF) values for all retained features were listed in Table S2. NYHA functional class was reported as four categories (I–IV) in the baseline characteristics for descriptive purposes, whereas in all modeling analyses—including feature selection, model training, and SHAP interpretation—it was entered as a binary variable (III/IV vs. I/II).

**TABLE 2:** Univariate and multivariate analysis for mortality risk prediction.

Variable	Univariate			Multivariate		
	HR	95% CI	*p* value	HR	95% CI	*p* value
Sex	0.94	0.79–1.14	0.533			
Age	1.05	1.04–1.06	<0.001	1.04	1.03–1.04	<0.001
BMI	0.93	0.90–0.96	<0.001	0.96	0.94–0.99	0.007
SBP	0.99	0.99–1.00	0.003	0.99	0.99–1.00	0.029
DBP	0.99	0.98–0.99	<0.001			
HR	1.01	1.00–1.01	0.034			
Hb	0.98	0.98–0.98	<0.001	0.99	0.99–1.00	<0.001
Cr	1.00	1.00–1.00	<0.001	1.00	1.00–1.00	0.070
Alb	0.92	0.90–0.94	<0.001	0.98	0.96–1.00	0.030
Tbil	1.01	1.00–1.01	<0.001	1.01	1.00–1.01	0.003
LVEF	0.97	0.97–0.98	<0.001	0.99	0.98–0.99	<0.001
LVEDD	1.02	1.01–1.03	<0.001			
LA	1.01	1.00–1.02	0.024			
NYHA (III/IV vs. I/II)	3.26	2.65–4.02	<0.001	1.89	1.50–2.37	<0.001
Smoking	0.92	0.82–1.03	0.149			
HBP	1.26	1.04–1.52	0.016			
Hyperlipidemia	1.20	0.92–1.56	0.173			
DM	1.66	1.34–2.06	<0.001			
CAD	1.63	1.35–1.96	<0.001			
MI	1.76	1.39–2.22	<0.001			
AF	1.29	1.06–1.57	0.010			
PAD	1.38	0.81–2.35	0.238			
Stroke	1.54	1.17–2.02	0.002			
COPD	2.00	1.47–2.72	<0.001			
CKD	3.85	3.07–4.84	<0.001	1.93	1.47–2.53	<0.001
Cancer	3.58	2.31–5.55	<0.001	2.25	1.45–3.51	<0.001
Cardiomyopathy	1.64	1.28–2.10	<0.001			
PAH	1.85	1.43–2.24	<0.001			
Etiology	1.18	0.98–1.43	0.077			
Severe MR	1.03	0.85–1.25	0.743			
Significant AR	1.33	1.05–1.67	0.017			
Significant AS	1.83	1.27–2.62	0.001	1.55	1.07–2.24	0.021
Significant MS	0.52	0.33–0.80	0.003			
Significant TR	1.72	1.43–2.08	<0.001			
PCI	0.96	0.74–1.24	0.726			
CABG	0.54	0.32–0.92	0.023			
PM	1.49	1.10–2.02	0.010			
Surgery	0.16	0.10–0.26	<0.001	0.29	0.18–0.48	<0.001
Transcatheter	0.00	0.00–Inf	0.987			
Beta‐blocker	0.85	0.71–1.03	0.103			
Diuretics	2.03	1.53–2.71	<0.001	1.67	1.23–2.27	<0.001
ACEI/ARB	0.71	0.59–0.86	<0.001	0.63	0.52–0.77	<0.001

Abbreviations: ACEI/ARB, angiotensin‐converting enzyme inhibitor/angiotensin receptor blocker; AF, atrial fibrillation; Alb, albumin; AR, aortic regurgitation; AS, aortic stenosis; BMI, body mass index; CABG, coronary artery bypass grafting; CAD, coronary artery disease; CI, confidence interval; CKD, chronic kidney disease; COPD, chronic obstructive pulmonary disease; Cr, creatinine; DBP, diastolic blood pressure; DM, diabetes mellitus; eGFR, estimated glomerular filtration rate; Hb, hemoglobin; HBP, hypertension; HR (clinical variable), heart rate; HR, hazard ratio; LA, left atrium diameter; LVEDD, left ventricular end‐diastolic diameter; LVEF, left ventricular ejection fraction; MI, myocardial infarction; MR, mitral regurgitation; MS, mitral stenosis; NYHA, New York Heart Association functional class; PAD, peripheral arterial disease; PAH, pulmonary arterial hypertension; PCI, percutaneous coronary intervention; PM, pacemaker; SBP, systolic blood pressure; Tbil, total bilirubin; TR, tricuspid regurgitation.

### 5.5 ML Model Construction

To identify the optimal algorithm for predicting mortality risk in patients with MR, 17 ML algorithms and traditional statistical models were trained and compared in the training set. Specifically, these included (1) ten algorithms implemented via the caret package of the R software: the Random Forest, Gradient Boosting (xgbTree implementation), Support Vector Machine with Radial Kernel, *K*‐Nearest Neighbors, Partial Least Squares, Gradient Boosting Machine (gbm implementation), Neural Network, Naïve Bayes, Linear Discriminant Analysis, and AdaBoost. (2) The CatBoost (Categorical Boosting), implemented using the catboost R package. This algorithm represents an advanced gradient boosting decision tree ensemble method that employs ordered boosting and gradient bias correction mechanisms, effectively reducing overfitting risk while automatically handling categorical variables. (3) The LightGBM (Light Gradient Boosting Machine), implemented using the lightgbm R package. This algorithm constitutes an efficient gradient boosting framework utilizing histogram‐based decision tree algorithms and leaf‐wise splitting strategies, achieving substantial improvements in training speed and memory efficiency while maintaining predictive accuracy. (4) The conventional Logistic Regression and Cox proportional hazards model; (5) the CoxScore, an integer risk scoring system based on HRs derived from the Cox model; (6) the published MitraScore [11] as a benchmark comparator; (7) the EuroSCORE II [29], a widely adopted clinical risk prediction model for mortality after cardiac surgery, was included as an additional benchmark comparator. Due to lack of the variable of poor mobility, EuroSCORE II was calculated without this predictor. Models were trained using the 15 predictive variables identified during the feature selection phase. Hyperparameters for each algorithm were optimized using 5‐fold cross‐validation on the training set. The final models were trained on the entire training set with optimal hyperparameters and evaluated on an independent validation set. Detailed parameter grid specifications are provided in Table S3. All models output predicted probabilities of mortality during follow‐up, ranging from 0 to 1. The optimal classification threshold was determined in the training set using Youden index maximization and was consistently applied to both internal and external validation sets.

### 5.6 SHAP Interpretability Analysis

To enhance clinical interpretability of the optimal model, the SHAP framework was adopted to explain model predictions. The SHAP values quantified the impact of specific features on individual patient predictions in the training set relative to baseline predicted values. All predictive variables were ranked in descending order by mean absolute SHAP values, generating a feature importance bar plot. A SHAP bee swarm plot was constructed to visualize the relationship between feature values and their impact on predictions. To gain deeper insights into the mechanisms of key predictive variables, SHAP dependence plots were generated for the top six most important features. These plots illustrated the relationship between specific feature values and their corresponding SHAP values, revealing potential nonlinear functional relationships between features and predicted outcomes. Individual‐level SHAP waterfall plots and force plots were generated for representative cases to provide personalized explanations.

### 5.7 Statistical Analysis

Continuous variables were presented as median and interquartile range (IQR) or mean ± standard deviation (SD) according to data distribution. Categorical variables were reported as counts and percentages. Baseline characteristics were compared between training and validation cohorts using the Mann–Whitney *U* test or Student's *t*‐test for continuous variables and the chi‐square test or Fisher's exact test for categorical variables, as appropriate; comparisons among the training, internal validation, and external validation sets were performed using one‐way analysis of variance or the Kruskal–Wallis test for continuous variables and the chi‐square test for categorical variables. Missing data were handled using multiple imputation by chained equations (Tables S4 and S5). Univariate Cox proportional hazards regression was performed to evaluate the association between each candidate variable and all‐cause mortality. Variables with *p* < 0.05 in univariate analysis were entered into multivariable Cox regression. The proportional hazards assumption was verified using Schoenfeld residuals.

Model discrimination was assessed using the area under the receiver operating characteristic curve (AUC) with 95% CIs calculated using DeLong's method. Comparisons of AUC between models were performed using DeLong's test. Sensitivity, specificity, positive predictive value (PPV), negative predictive value (NPV), and *F*1 score were calculated on the basis of the optimal threshold determined by Youden index maximization. Model calibration was evaluated using calibration curves and Brier Scores. Clinical utility was assessed through decision curve analysis and clinical impact curves. Confusion matrices were employed to visualize classification results, displaying the distribution of true positives, false positives, true negatives, and false negatives. Residual plots (distribution of differences between predicted probabilities and actual outcomes) were used to assess patterns of prediction errors and identify potential systematic biases.

Patients were stratified into high‐risk and low‐risk groups on the basis of model‐predicted probabilities and the optimal threshold. Kaplan–Meier curves were constructed to visualize survival differences, with statistical comparison performed using the log‐rank test. Multivariable Cox proportional hazards regression was employed to calculate adjusted HRs and 95% CI for the high‐risk group relative to the low‐risk group, adjusting for potential confounders. Subgroup analyses were conducted to evaluate model performance across clinically relevant patient subsets: (1) primary versus secondary MR; (2) NYHA functional class II‐IV; (3) LVEF ≤ 60%; and (4) conservative treatment. The AUC with 95% CI was calculated for each subgroup. Interaction tests were performed to assess whether predictive effects varied across subgroups.

All statistical analyses were performed using R version 4.1.0 (R Foundation for Statistical Computing, Vienna, Austria). All statistical tests were two‐sided, and *p* < 0.05 was considered statistically significant. Statistical figures were generated using R version 4.3.2 and assembled in Adobe Illustrator version 26.0 (Adobe Inc., San Jose, CA, USA). This study is reported in accordance with the TRIPOD+AI statement, and the completed checklist is provided as a separate file.

## Author Contributions

**Yuzhe Yin**: methodology, formal analysis, data curation, investigation, writing – original draft, writing – review and editing. **Junxing Lv**: methodology, formal analysis, data curation, investigation, writing – original draft, writing – review and editing. **Qinghao Zhao**: methodology, formal analysis, data curation, investigation, writing – original draft, writing – review and editing. **Yunqing Ye**: data curation, investigation, writing – review and editing. **Weiwei Wang**: data curation, investigation, writing – review and editing. **Haitong Zhang**: data curation, investigation, writing – review and editing. **Zhenyan Zhao**: data curation, investigation, writing – review and editing. **Qingrong Liu**: data curation, investigation, writing – review and editing. **Zikai Yu**: data curation, investigation, writing – review and editing. **Bin Zhang**: data curation, investigation, writing – review and editing. **Shuai Guo**: data curation, investigation, writing – review and editing. **Zhenya Duan**: data curation, investigation, writing – review and editing. **Bincheng Wang**: data curation, investigation, writing – review and editing. **Yanyan Zhao**: data curation, investigation, writing – review and editing. **Zhe Li**: data curation, investigation, writing – review and editing. **Runlin Gao**: conceptualization, supervision, data curation, investigation, writing – review and editing. **Haiyan Xu**: conceptualization, supervision, data curation, investigation, writing – review and editing. **Yongjian Wu**: conceptualization, supervision, data curation, investigation, writing – review and editing. **Junbo Ge**: conceptualization, supervision, data curation, investigation, writing – review and editing. All authors read and approved the final manuscript.

## Funding

This work was supported by the Postdoctoral Fellowship Program of CPSF (Grant GZC20261196), the Chinese Academy of Medical Sciences Innovation Fund for Medical Sciences (Grant 2017‐I2M‐3‐002), and the Chinese Academy of Medical Sciences Innovation Fund for Medical Sciences (Grant 2025‐I2M‐C&T‐B‐026).

## Ethics Statement

The China‐VHD registry (approval no. 2017‐968; ClinicalTrials.gov identifier NCT03484806) and the China‐DVD registry (approval no. 2016‐777; ClinicalTrials.gov identifier NCT02865798) were approved by the institutional review boards or ethics committees of all participating centers. Both studies were performed in accordance with the principles of the Declaration of Helsinki.

## Consent

All participants provided written informed consent before enrollment.

## Patient and Public Involvement

Patients or the public were not involved directly in the design, conduct, reporting, or dissemination plans of our study.

## Conflicts of Interest

The authors declare no conflicts of interest.

## Research Registration Unique Identification Number (UIN)

This study is based on two nationally registered prospective cohorts: China‐VHD registry (NCT03484806) and China‐DVD registry (NCT02865798).

## Supporting information




**Supporting File**: mco271005‐sup‐0001‐SuppMat.docx.

## Data Availability

The data used during the current study are available from the corresponding authors on reasonable request.
