# Supplementary material for: Machine Learning‐Based Prediction of Mortality in Patients With Mitral Regurgitation: Results From Two Nationwide, Prospective Studies
Source: MedComm (2020). 2026 Oct 9;7(10):e71005. doi: 10.1002/mco2.71005 (PMC13650608; doi:10.1002/mco2.71005)
Supplement: Supplementary file 1 [file MCO2-7-e71005-s001.docx]

**Machine Learning-Based Prediction of Mortality in Patients with Mitral Regurgitation: Results from Two Nationwide, Prospective Studies**

**Supplementary Information**

Yuzhe Yin, Junxing Lv, Qinghao Zhao, Yunqing Ye, Weiwei Wang, Haitong Zhang, Zhenyan Zhao, Qingrong Liu, Zikai Yu, Bin Zhang, Shuai Guo, Zhenya Duan, Bincheng Wang, Yanyan Zhao, Runlin Gao, Zhe Li, Haiyan Xu, Yongjian Wu, and Junbo Ge, for the CHINA-VHD collaborators

Affiliations and corresponding-author details are identical to those in the main manuscript.

**I. Supplementary Methods.**

**The echocardiographic definition of significant MR in the China-VHD study**

**Significant MR:** vena contracta ≥0.3cm, or the central jet MR>20% left atrium, or an effective regurgitation orifice ≥0.2cm^2^, or a regurgitant volume ≥30mL/beat.

**Severe MR:** vena contracta ≥0.7cm, or the central jet MR>40% left atrium, or an effective regurgitation orifice ≥0.2cm^2^ (for secondary MR) or ≥0.4cm^2^ (for primary MR), or a regurgitant volume ≥30mL/beat (for secondary MR) or ≥60mL/beat (for primary MR), or regurgitant jet reaching the posterior wall or the top of the left atrium.

2. **Supplementary Tables**

**Table S1. Three Methods Variable Screening Results**

| Variables | Cox Stepwise Regression | Cox-LASSO (1se) | Boruta Algorithm | Intersection of All Methods |
| --- | --- | --- | --- | --- |
| sex |  |  | √ |  |
| age | √ | √ | √ | √ |
| BMI | √ | √ | √ | √ |
| SBP | √ | √ | √ | √ |
| DBP |  |  | √ |  |
| HR |  |  | √ |  |
| Hb | √ | √ | √ | √ |
| Cr | √ | √ | √ | √ |
| Alb | √ | √ | √ | √ |
| Tbil | √ | √ | √ | √ |
| LVEF | √ | √ | √ | √ |
| LVEDD | √ |  |  |  |
| LA | √ |  |  |  |
| NYHA (III/IV vs. I/II) | √ | √ | √ | √ |
| Smoking |  |  |  |  |
| HBP |  |  | √ |  |
| Hyperlipidemia |  |  |  |  |
| DM |  |  | √ |  |
| CAD | √ |  |  |  |
| MI | √ |  | √ |  |
| AF |  |  |  |  |
| PAD |  |  |  |  |
| Stroke |  |  |  |  |
| COPD |  |  |  |  |
| CKD | √ | √ | √ | √ |
| Cancer | √ | √ | √ | √ |
| Cardiomyopathy |  |  |  |  |
| PAH | √ |  |  |  |
| Etiology | √ |  | √ |  |
| Severe MR |  |  |  |  |
| Significant AR |  |  |  |  |
| Significant AS | √ | √ | √ | √ |
| Significant MS |  |  |  |  |
| Significant TR | √ |  |  |  |
| PCI | √ |  | √ |  |
| CABG | √ |  |  |  |
| PM |  |  |  |  |
| Surgery | √ | √ | √ | √ |
| Transcatheter | √ |  |  |  |
| Betablocker | √ |  | √ |  |
| Diuretics | √ | √ | √ | √ |
| ACEI/ARB | √ | √ | √ | √ |

Abbreviations: BMI, body mass index; SBP, systolic blood pressure; DBP, diastolic blood pressure; HR (clinical variable), heart rate; Hb, hemoglobin; Cr, creatinine; Alb, albumin; Tbil, total bilirubin; eGFR, estimated glomerular filtration rate; LVEF, left ventricular ejection fraction; LVEDD, left ventricular end-diastolic diameter; LA, left atrium diameter; NYHA, New York Heart Association functional class; HBP, hypertension; DM, Diabetes mellitus; CAD, coronary artery disease; MI, myocardial infarction; AF, atrial fibrillation; PAD, peripheral arterial disease; COPD, chronic obstructive pulmonary disease; CKD, chronic kidney disease; PAH, pulmonary arterial hypertension; MR, mitral regurgitation; AR, aortic regurgitation; AS, aortic stenosis; MS, mitral stenosis; TR, tricuspid regurgitation; PCI, percutaneous coronary intervention; CABG, coronary artery bypass grafting; PM, pacemaker; ACEI/ARB, angiotensin-converting enzyme inhibitor/angiotensin receptor blocker.

**Table S2.** Variance Inflation Factor (VIF) values for all retained features

| Variable | VIF |
| --- | --- |
| ACEIARB | 1.093 |
| Age | 1.188 |
| Alb | 1.183 |
| BMI | 1.060 |
| Cancer | 1.017 |
| CKD | 1.297 |
| Cr | 1.335 |
| Tbil | 1.083 |
| Hb | 1.238 |
| NYHA | 1.312 |
| Surgery | 1.362 |
| AS | 1.014 |
| SBP | 1.115 |
| LVEF | 1.449 |
| Diuretics | 1.091 |

*Note: VIF < 5 indicates no multicollinearity concern.*

**Table S3. Parameters for models.**

| Model | Parameter | Value |
| --- | --- | --- |
| RandomForest | mtry | 2 |
| GradientBoosting | nrounds | 100 |
| GradientBoosting | max_depth | 3 |
| GradientBoosting | eta | 0.1 |
| GradientBoosting | gamma | 0 |
| GradientBoosting | colsample_bytree | 0.7 |
| GradientBoosting | min_child_weight | 1 |
| GradientBoosting | subsample | 0.7 |
| SVM_Kernel | sigma | 0.1 |
| SVM_Kernel | C | 0.5 |
| LogisticModel | parameter | none |
| NeighborMethod | k | 15 |
| PLSModel | ncomp | 2 |
| BoostingMethod | n.trees | 50 |
| BoostingMethod | interaction.depth | 1 |
| BoostingMethod | shrinkage | 0.1 |
| BoostingMethod | n.minobsinnode | 5 |
| NeuralNet | size | 3 |
| NeuralNet | decay | 0 |
| BayesMethod | fL | 0 |
| BayesMethod | usekernel | TRUE |
| BayesMethod | adjust | 1 |
| DiscriminantModel | parameter | none |
| AdaptiveBoosting | mfinal | 50 |
| AdaptiveBoosting | maxdepth | 1 |
| AdaptiveBoosting | coeflearn | Breiman |
| CATBoost | depth | 4 |
| CATBoost | learning_rate | 0.1 |
| CATBoost | l2_leaf_reg | 5 |
| CATBoost | iterations | 52 |
| LightGBM | num_leaves | 15 |
| LightGBM | learning_rate | 0.05 |
| LightGBM | min_data_in_leaf | 20 |
| LightGBM | lambda_l2 | 0 |
| LightGBM | nrounds | 36 |

**Table S4. Missing Data and Imputation Methods in the China-VHD Cohort**

| Variable | Missing (n) | Missing (%) | Imputation Method |
| --- | --- | --- | --- |
| BMI | 178 | 2.8 | Predictive mean matching |
| NYHA | 2 | 0.03 | Predictive mean matching |
| SBP | 2 | 0.03 | Predictive mean matching |
| DBP | 2 | 0.03 | Predictive mean matching |
| HR | 2 | 0.03 | Predictive mean matching |
| Hb | 524 | 8.3 | Predictive mean matching |
| Cr | 519 | 8.2 | Predictive mean matching |
| Alb | 628 | 9.9 | Predictive mean matching |
| Tbil | 701 | 11.1 | Predictive mean matching |
| LVEF | 1 | 0.02 | Predictive mean matching |

BMI, body mass index; NYHA, New York Heart Association functional class; SBP, systolic blood pressure; DBP, diastolic blood pressure; HR, heart rate; Hb, hemoglobin; Cr, serum creatinine; Alb, serum albumin; Tbil, total bilirubin; LVEF, left ventricular ejection fraction.

**Table S5. Missing Data and Imputation Methods in the China-DVD Cohort**

| Variable | Missing (n) | Missing (%) | Imputation Method |
| --- | --- | --- | --- |
| NYHA | 577 | 12.4 | Predictive mean matching |
| Cr | 199 | 4.3 | Predictive mean matching |
| Alb | 293 | 6.3 | Predictive mean matching |
| Tbil | 125 | 2.7 | Predictive mean matching |
| LVEDD | 123 | 2.6 | Predictive mean matching |
| LA | 114 | 2.4 | Predictive mean matching |
| Etiology | 58 | 1.2 | Logistic regression |
| Hb | 24 | 0.5 | Predictive mean matching |
| DBP | 14 | 0.3 | Predictive mean matching |
| HR | 14 | 0.3 | Predictive mean matching |
| SBP | 12 | 0.3 | Predictive mean matching |
| TR | 12 | 0.3 | Logistic regression |
| AR | 5 | 0.1 | Logistic regression |
| LVEF | 3 | 0.1 | Predictive mean matching |

NYHA, New York Heart Association functional class; Cr, serum creatinine; Alb, serum albumin; Tbil, total bilirubin; LVEDD, left ventricular end-diastolic diameter; LA, left atrial diameter; Hb, hemoglobin; DBP, diastolic blood pressure; HR, heart rate; SBP, systolic blood pressure; TR, tricuspid regurgitation; AR, aortic regurgitation; LVEF, left ventricular ejection fraction.

**Table S6.** Brier Score comparison across all prediction models

| Model | Training Set | Internal Validation | External Validation |
| --- | --- | --- | --- |
| **CatBoost** | **0.0814** | **0.0685** | **0.0875** |
| LightGBM | 0.0823 | 0.0473 | 0.0893 |
| Random Forest | 0.0817 | 0.0812 | 0.0870 |
| Gradient Boosting | 0.0823 | 0.0827 | 0.0897 |
| Boosting Method | 0.0825 | 0.0816 | 0.0865 |
| Adaptive Boosting | 0.1086 | 0.1053 | 0.1280 |
| Logistic Regression | 0.0813 | 0.0790 | 0.0883 |
| SVM Kernel | 0.0885 | 0.0863 | 0.0917 |
| Neural Network | 0.0872 | 0.0858 | 0.0880 |
| K-Nearest Neighbor | 0.0871 | 0.0870 | 0.0913 |
| PLS Model | 0.1290 | 0.1302 | 0.1362 |
| Naive Bayes | 0.0937 | 0.0939 | 0.0958 |
| Discriminant Model | 0.0830 | 0.0811 | 0.0937 |
| Cox Regression | 0.0852 | 0.0823 | 0.1041 |
| EuroSCORE II | 0.0931 | 0.0937 | 0.0914 |
| CoxScore | 0.3679 | 0.3827 | 0.4431 |
| MitraScore | 0.3669 | 0.3795 | 0.4226 |

*The Brier Score measures the mean squared difference between predicted probabilities and observed outcomes, ranging from 0 (perfect) to 1 (worst). Lower values indicate better calibration and overall predictive accuracy.*

**Table S7.** Bayesian Information Criterion (BIC) and DeLong test comparison between CatBoost and Cox models

| Metric | Training Set | Internal Validation | External Validation |
| --- | --- | --- | --- |
| BIC |  |  |  |
| CatBoost | 2260.62 | 1173.50 | 2979.32 |
| Cox | 2663.91 | 1224.84 | 3490.69 |
| ΔBIC (CatBoost − Cox) | -403.29 | -51.34 | -511.37 |
| DeLong Test |  |  |  |
| Z / D statistic | 10.691 | 2.382 | 0.233 |
| P value | <0.001 | 0.017 | 0.816 |

*Abbreviations: BIC, Bayesian Information Criterion; ΔBIC, difference in BIC between CatBoost and Cox models. A negative ΔBIC indicates superior model fit for CatBoost. ΔBIC < −10 is generally considered strong evidence favoring the model with lower BIC.*

*BIC = −2 × log-likelihood + k × ln(n), where k is the number of model parameters and n is the sample size.*

*DeLong test compares the AUCs of CatBoost and Cox models. A P value < 0.05 indicates a statistically significant difference in discriminative performance between the two models.*

**3. Supplementary Figures.**

Figure S1. Reverse cumulative residual distributions and residual boxplots for the training, internal validation, and external validation datasets.

Figure S2. Confusion matrices in the training, internal validation, and external validation datasets. (A) Training set; (B) Internal validation set; (C) External validation set.

Figure S3. SHAP feature dependence plots for the best-performing model.

Figure S4. SHAP waterfall plot and force plot for two representative cases based on the best-performing predictive model.

Figure S5. ROC curves of the best-performing model in selected patient subsets within the internal and external validation datasets. A. Primary Mitral Regurgitation; B. Secondary Mitral Regurgitation; C. NYHA Functional Class II–IV; D. Left Ventricular Ejection Fraction ≤60%; E. No In-Hospital Valve Intervention

Figure S6. Forest plot for subgroup analysis.

Figure S7. ROC curves and calibration plots of the full-variable CatBoost model in the training, internal validation, and external validation datasets.

Figure S8. SHAP value distribution of the full-variable CatBoost model showing feature importance and direction of effect across the study population.

Figure S9. Time-dependent ROC curves at 6- and 12-month of the CatBoost model in the training, internal validation, and external validation datasets.

**Figure S10. Web interface of the MitraBoost online calculator with representative input–output examples.**
Screenshots of the publicly available MitraBoost application deployed on Hugging Face Spaces (<https://huggingface.co/spaces/YuzheYin/MitraBoost>). For each case, the sidebar panel shows the 15 input variables entered, and the result panel displays the model-predicted mortality probability together with the corresponding risk classification (high vs. low risk, based on the predefined threshold of 0.103). **(A)** Patient 1; **(B)** Patient 2.
